# Lipase-Catalyzed Kinetic Resolution of Aryltrimethylsilyl Chiral Alcohols

**DOI:** 10.3390/molecules16119697

**Published:** 2011-11-23

**Authors:** Dayvson J. Palmeira, Juliana C. Abreu, Leandro H. Andrade

**Affiliations:** Institute of Chemistry, University of São Paulo, Av. Prof. Lineu Prestes, n°. 748, SP 05508-900, São Paulo, Brazil; Email: dayvson.palmeira@gmail.com (D.J.P.); jukinhabreu@hotmail.com (J.C.A.)

**Keywords:** lipase, aryltrimethylsilyl chiral alcohols, kinetic resolution, transesterification

## Abstract

Lipase-catalyzed kinetic resolution of aryltrimethylsilyl chiral alcohols through a transesterification reaction was studied. The optimal conditions found for the kinetic resolution of *m*- and *p*-aryltrimethylsilyl chiral alcohols, led to excellent results, high conversions (*c* = 50%), high enantiomeric ratios (*E* > 200) and enantiomeric excesses for the remaining (*S*)-alcohol and (*R*)-acetylated product (>99%). However, kinetic resolution of *o*-aryltrimethylsilyl chiral alcohols did not occur under the same conditions applied to the other isomers.

## 1. Introduction

The chemistry of organosilicon compounds is well-established nowadays due to its importance in organic synthesis, in which these compounds can be used for carbon-carbon bond formation as well as protecting-group reagents [1,2]. In addition, most of the organosilicon compounds are generally easy to handle and store. They are thermal stable and present low toxicity [3,4]. These characteristics make the organosilicon compounds a good choice for diverse organic synthesis methodologies. Aryltrimethylsilyl compounds have attracted special attention because they are involved in some interesting desilylative functionalizations, such as aryne formation [5,6,7,8,9,10], oxyarylation of alkenes [11,12], phosphazene base-promoted functionalization [13], metal-catalyzed cross-coupling reactions [14,15,16,17,18] and palladium-catalyzed oxidative coupling reaction [19].

Although silicon-containing organic compounds find a remarkable application in organic synthesis, there is a general lack of biocatalyzed studies using these compounds as substrates, and only a few such biocatalyzed transformations of organosilanes have been reported, such as the stereoselective microbial reduction of racemic acetyl(*t*-butyl)methylphenyl silane [20], preparation of *p-*(trimethyl- silyl)phenylalanine through an amidohydrolase produced by a bacterium [21], enantioselective microbial reduction of acyl silanes [22], the synthesis of (*R*)-2-trimethylsilyl-2-hydroxyethylcyanide by a (*R*)-oxynitrilase-catalyzed hydrogen cyanide addition [23], biocatalytic cleavage of silicon-oxygen bonds [24,25] and enzyme-catalyzed siloxane bond formation [26,27,28]. Reports on lipase-catalyzed kinetic resolution of organosilanes are even scarcer. To our knowledge, just a few publications have focused on this type of biocatalytic reaction, such as the synthesis of optically active 2-sila-1,3-propanediols through a regioselective transesterification reaction catalyzed by a series of lipases [29], the preparation of optically active silylmethanol derivatives through lipase-catalyzed enantioselective esterification and transesterification [30], enantioselective transesterification of 1-trimethylsilyl-1-alkyn-3-ols catalyzed by immobilized *Pseudomonas cepacia* lipase PS and *Pseudomonas fluorescens *[31,32,33], and the kinetic resolution of 4-(phenyldimethylsilyl)-3-butyn-2-ol for the preparation of enantioenriched allenylsilanes [34]. However, there are no reports in the literature, in which aryltrimethylsilyl compounds have been used in lipase-catalyzed kinetic resolution. The aim of this work was therefore to carry out an extensive study on the lipase-catalyzed kinetic resolution of aryltrimethylsilyl chiral alcohols *via* transesterification reaction.

## 2. Results and Discussion

### 2.1. Synthesis of Aryltrimethylsilyl Chiral Alcohols ***2a–f***

**Scheme 1:**
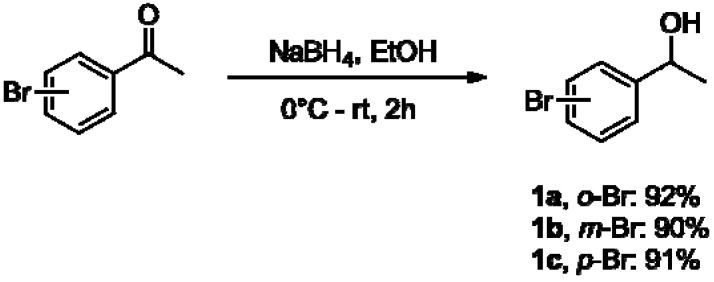
Reduction of bromo-substituted acetophenones to afford chiral alcohols **1a–c**.

1-(Bromo-phenyl)ethanols **1a–f** were synthesized in excellent yields (90–92%) by reacting *o*-, *m*-, and *p*-bromo-substituted acetophenones with NaBH_4_ (Scheme 1) [35].

**Scheme 2:**
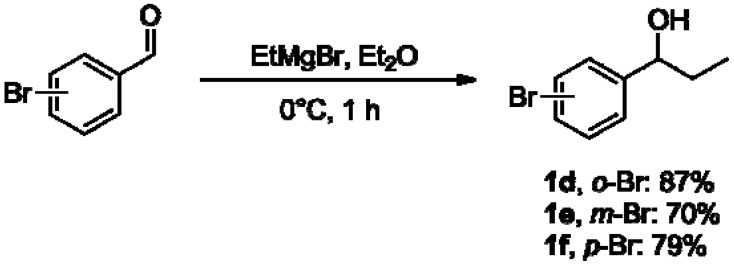
Carbonyl addition of a Grignard reagent to bromo-substituted benzaldehydes to give chiral alcohols **1d–f**.

The addition of a Grignard reagent to the *o-*, *m-*, and *p-*bromo-substituted benzaldehydes gave the chiral alcohols **1d–f** bearing an ethyl group attached at the benzylic carbon, in good yields (70%–87%, Scheme 2).

**Scheme 3:**
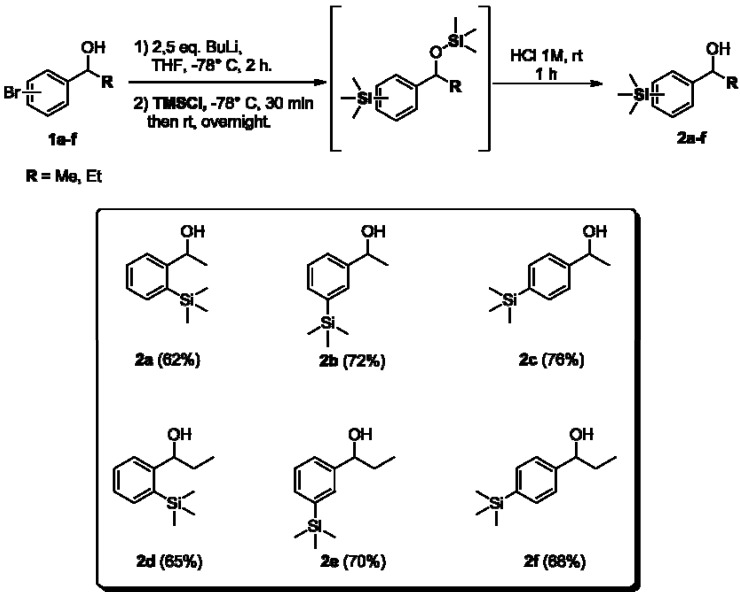
Synthesis of the aryltrimethylsilyl chiral alcohols **2a–f**.

With the aryl bromides precursors **1a–f** in hand, we next synthesized aryltrimethylsilyl chiral alcohols. The compounds **1a–f** were submitted to the bromo-lithium exchange reaction with *n*-butyl-lithium, THF at −70 °C, followed by addition of trimethylsilyl chloride. The disilylated intermediates were generated *in situ*, and after acid hydrolysis reaction, aryltrimethylsilyl chiral alcohols **2a–f** were obtained in moderate to good yields (62%–76%, Scheme 3).

### 2.2. Enzymatic Kinetic Resolution of the Aryltrimethylsilyl Chiral Alcohols ***2a–f***

Initially, we focused our study on choosing the best lipase source for the enzymatic kinetic resolution (EKR) of aryltrimethylsilyl chiral alcohols through transesterification reaction. For the lipase screening, vinyl acetate was chosen as the acyl donor, hexane as the solvent, and the compound (*RS*)-**2c** as the model substrate. Eleven types of lipase sources were used in the kinetic resolution of compound (*RS*)-**2c**. The results are summarized in Table 1.

**Table 1:** Screening of lipases for the kinetic resolution of (*RS*)-**2c**^ a^.

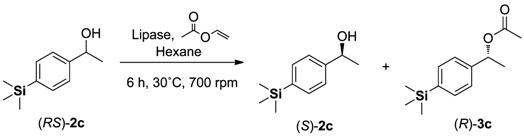					
Entry	Lipase (source)	*ee *(%) ^b^		*c* (%) ^c^	*E * ^d^
		(*S*)-2c	(*R*)-3c		
1	*Mucor javanicus* (Amano M)	–	–	–	–
2	*Candida cylindracea* (Fluka)	–	–	–	–
3	*Candida antarctica* (CAL-B, Novozym 435)	74	>99	43	>200
4	*Pseudomonas cepacia* (Amano PS)	48	>99	33	>200
5	*Aspergillus niger* (Amano A)	–	–	–	–
6	*Pseudomonas cepacia* (Amano PS-D I)	98	>99	49	>200
7	*Candida rugosa* (Amano, type VII)	–	–	–	–
8	*Pseudomonas cepacia* (Amano PS-C II)	>99	>99	50	>200
9	*Mucor meihei* (Sigma)	–	–	–	–
10	*Pseudomonas fluorescens* (Amano AK)	58	>99	37	>200
11	Porcine pancreas (Sigma, type II)	–	–	–	–

^a^ General conditions: Substrate (0.05 mmol), lipase (2 mg), vinyl acetate (0.11 mmol), hexane (200 µL); ^b^ Determined by chiral GC analysis; ^c^ Conversion: *c* = *ee*_S_/(*ee*_S_ + *ee*_P_); ^d^* E* = ln{[*ee*_P_(1 − *ee*_S_)/(*ee*_P_ + *ee*_S_)]}/ln{[*ee*_P_(1 + *ee*_S_)/(*ee*_P_ + *ee*_S_)]}; (–) = No reaction.

Lipases from *Mucor javanicus*, *Candida cylindracea*, *Aspergillus niger*, *Candida rugosa*, *Mucor meihei* and porcine pancreas (Table 1, entries 1, 2, 5, 7, 9 and 11) showed no activity for the transesterification reaction of (*RS*)-**2c**, and only unreacted racemic alcohol was recovered at the end of the process. On the other hand, high enantiomeric ratios (*E *> 200) and excellent enantiomeric excess for (*R*)-**3c** (*ee* > 99%) were achieved when lipases from *Candida antarctica *(CAL-B), *Pseudomonas cepacia* [free form (Amano PS), immobilized on diatomite (Amano PS-DI) or immobilized on ceramic (Amano PS-CII)] and *Pseudomonas fluorescens* (entries 3, 4, 6, 8 and 10) were used as biocatalysts. When *Pseudomonas cepacia* immobilized on diatomite (Amano PS-DI) or ceramic (Amano PS-CII) (entries 6 and 8) were used, high conversions were obtained (49% and 50%, respectively). Based on these results and aiming future application in the chemoenzymatic dynamic resolution of aryltrimethylsilyl chiral alcohols, the lipase from *Pseudomonas cepacia* Amano PS-C II (Table 1, entry 8) was chosen for the subsequent studies. The amount of enzyme and reaction time were also evaluated in order to achieve the perfect kinetic resolution process (Table 2).

**Table 2:** Evaluation of reaction time and enzyme/substrate ratio for kinetic resolution of (*RS*)-**2c**^ a^.

Entry	Substrate (mmol)	Amano PS-C II (mg)	Time (h)	*ee *(%) ^b^		*c* (%) ^c^	*E * ^d^
				(*S*)-2c	(*R*)-3c		
1	0.05	2	6	>99	>99	50	>200
2	0.05	2	3	>99	>99	50	>200
3	0.05	1	6	>99	>99	50	>200
4	0.05	1	3	>99	>99	50	>200
5	0.05	1	1.5	86	>99	46	>200
6	0.1	1	6	>99	>99	50	>200
7	0.1	1	3	83	>99	45	>200
8	0.1	1	1.5	48	>99	33	>200

^a^ General conditions: vinyl acetate (2.2 equiv.), hexane (200 µL); ^b^ Determined by chiral GC analysis; ^c^ Conversion: *c* = *ee*_S_/(*ee*_S_ + *ee*_P_); ^d^* E* = ln{[*ee*_P_(1 − *ee*_S_)/(*ee*_P_ + *ee*_S_)]}/ln{[*ee*_P_(1 + *ee*_S_)/(*ee*_P_ + *ee*_S_)]}.

After 6 hours reaction, a decrease in the enzyme amount and an increase in substrate quantities (entries 1, 3 and 6) led to perfect kinetic resolution of (*RS*)-**2c**. However, excellent results were also achieved after 3 hours (entries 2 and 4), even with a decrease in enzyme concentration (entry 4). Maintaining the reaction time (3 h), 1 mg of enzyme, but increasing the amount of substrate (entry 7), a small decrease in conversion was observed. Reactions performed in 1.5 h (entries 5 and 8) presented the lowest conversions, indicating that the kinetic resolution of (*RS*)-**2c** did not occur completely.

Thus, the optimized conditions, considering reaction time and amount of substrate and enzyme (entry 4), was then applied to enzymatic kinetic resolution of several aryltrimethylsilyl chiral alcohols **2a**,**b**,**d**,**e**,**f** (Table 3).

The EKR of compounds (*R*,*S*)-**2b**,**f** (Table 3, entries 3 and 9, respectively) performed in 3 h led to very good results with high conversions (47% and 49%, respectively) and excellent enantioselectivity (*E* > 200 in both cases). We extended the reaction time to 16 h (Table 3, entries 4 and 10), and a perfect kinetic resolution of (*R*,*S*)-**2b**,**f** was achieved (conversion of 50% and enantiomeric excesses for (*S*)-**2** and (*R*)-**3** >99%, in both cases). In regards to compound (*R*,*S*)-**2e**, a three-hour reaction (Table 3, entry 7) led to low conversion (*c* = 35%). Only when the reaction time and enzyme amount were increased to 24 h and 2 mg, respectively (Table 3, entry 8), excellent conversion (*c* = 50%), enantioselectivity (*E* > 200) and enantiomeric excesses (*ee* > 99% for both (*S*)-**2e** and (*R*)-**3e**) were found.

**Table 3:** Enzymatic kinetic resolution of aryltrimethylsilyl chiral alcohols **2a**,**b**,**d**,**e**,**f**^ a^.

Entry	Substrate	Time (h)	*ee *(%) ^b^		*c* (%) ^c^	*E * ^d^
			(*S*)-2	(*R*)-3		
1	(*RS*)-**2a**	3	–	–	–	–
2	(*RS*)-**2a**	16	–	–	–	–
3	(*RS*)-**2b**	3	87	>99	47	>200
4	(*RS*)-**2b**	16	>99	>99	50	>200
5	(*RS*)-**2d**	3	–	–	–	–
6	(*RS*)-**2d**	16	–	–	–	–
7	(*RS*)-**2e**	3	54	>99	35	>200
8	(*RS*)-**2e^e^**	24	>99	>99	50	>200
9	(*RS*)-**2f**	3	96	>99	49	>200
10	(*RS*)-**2f**	16	>99	>99	50	>200

^a^ General conditions: substrate (0.05 mmol), enzyme (1 mg), vinyl acetate (0.11 mmol), hexane (200 µL); ^b^ Determined by chiral GC analysis; ^c^ Conversion: *c* = *ee*_S_/(*ee*_S_ + *ee*_P_); ^d^* E* = ln{[*ee*_P_ (1 − *ee*_S_)/(*ee*_P_ + *ee*_S_)]}/ln{[*ee*_P_(1 + *ee*_S_)/(*ee*_P_ + *ee*_S_)]}; ^e^ Amount of enzyme: 2 mg; (–) = No reaction.

As it can be seen, after three-hour reaction, *p*-trimethylsilyl-substituted phenyl chiral alcohols **2c**,**f** (Table 2, entry 4 and table 3, entry 9) showed better conversion than *m*-trimethylsilyl substituted analogues **2b**,**e** (Table 3, entries 3 and 7). The size of the alkyl chain (methyl: **2b**, ethyl: **2e**) attached to the chiral carbon has a small influence in the conversion of alcohols **2b**,**e** into esters **3b**,**e**. For example, the enzymatic kinetic resolution of alcohol **2b** presented 47% conversion whereas for the alcohol **2e** presented 35% (Table 3, entries 3 and 7). Nevertheless, in all cases excellent enantioselectivities were achieved.

**Scheme 4:**
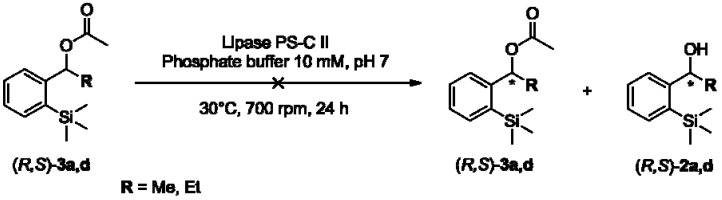
Attempts towards the enantioselective hydrolysis of (*RS*)-**3a**,**d**.

Lipase-catalyzed transesterification of *o*-trimethylsilyl substituted phenyl chiral alcohols (*RS*)-**2a**,**d** did not occur neither after 3 h (entries 1 and 5) nor 16 h (entries 2 and 6). To overcome this obstacle, attempts towards the enantioselective hydrolysis of (*RS*)-**3a**,**d** were made (Scheme 4) [36].

**Figure 1:**
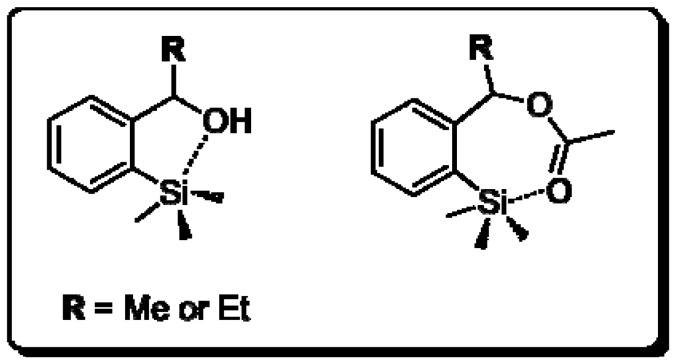
Possible pseudo-pentacoordinated species that can be present on the active site of the enzyme.

However, by using PS-C II lipase, phosphate buffer solution (10 mM, pH 7) and ester (*RS*)-**3a**,**d** as substrate, no hydrolysis products (**2a**,**d**) were obtained. A possible explanation for these results may involve a non-bonded Si**^…^**O attraction by pseudo-pentacoordinated species (Figure 1).

Pseudo-pentacoordinated species have been evidenced in studies involving silyl formates [37], di- and trisilanes bearing the (dimethylamino)-1-naphthyl group [38,39], *N*-substituted trimethylsilyl carbamates [40] and *N*-trimethylsilylated cyclic ureas [41]. Assuming the five- and seven-membered pseudo-rings formed from this intramolecular interaction, a steric hindrance, due to a rigid array of these structures, could be unfavorable for the formation of enzyme-substrate complexes.

This proposal is endorsed by the results obtained for the *m*- and *p*-trimethylsilyl substituted phenyl chiral alcohols, in which the non-bonded Si**^…^**O attraction should be less intense and pseudo-pentacoordinated species can hardly be formed. In these cases the kinetic resolution proceed as expected. Motivated by the excellent results, we decided to carry out the enzymatic kinetic resolution of (*R,S*)-**2b**,**c** and (*R,S*)-**2e**,**f** on a higher scale (Table 4).

**Table 4:** Lipase-catalyzed kinetic resolution of compounds (*R,S*)-**2b**,**c** and (*R,S*)-**2e**,**f**^ a^.

Entry	Substrate	Isolated yield % (*ee *%) ^b^	
1	(*RS*)-**2b**	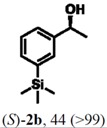	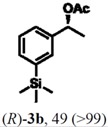
2	(*RS*)-**2c^c^**	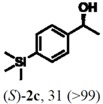	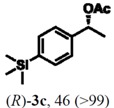
3	(*RS*)-**2e^d^**	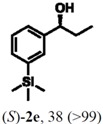	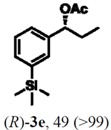
4	(*RS*)-**2f**	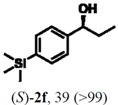	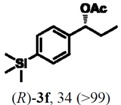

^a^ General conditions: substrate (1 mmol), enzyme (20 mg), vinyl acetate (2.2 mmol, 203 µL), hexane (4 mL), reaction time of 16 h; ^b^ Determined by chiral GC analysis; ^c^ Reaction time of 3 h; ^d^ Reaction time of 24 h and amount of enzyme = 40 mg.

According to the results on Table 4, the lipase-catalyzed kinetic resolution of (*R,S*)-**2b**,**c** and (*R,S*)-**2e**,**f** led to excellent results with enantiomeric excesses >99% in all cases and isolated yields of up to 49%.

### 2.3. Determination of Absolute Configuration of Enantioenriched Aryltrimethylsilyl Chiral Alcohols *(**2b**, **2c**, **2e** and **2f**)*

The enantioenriched compounds **2b**, **2c**, **2e** and **2f** were submitted to a bromodesilylation reaction in order to obtain the corresponding bromides (Scheme 5).

**Figure 5:**
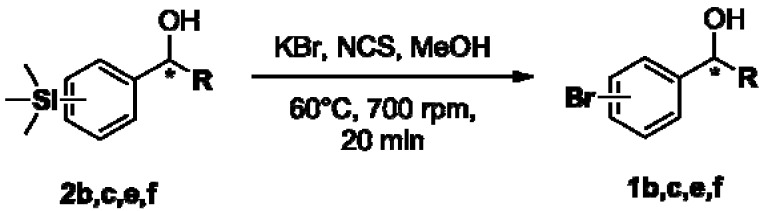
Bromodesilylation of enantioenriched compounds **2b**, **2c**, **2e** and **2f**.

The enantioenriched bromides **1b**, **1c**, **1e** and **1f** were submitted to chiral GC and HPLC analysis, and the retention times were compared with those described in the literature [42,43,44,45]. Thus, absolute configuration of enantioenriched aryltrimethylsilyl (*S*)-alcohols and (*R*)-acetates were determined by analogy to the results found for the bromophenyl (*S*)-alcohols. The stereochemistry found for the resolved aryltrimethylsilyl chiral alcohols and acetates is in accordance with the Kaslauskas’ rule prediction [46].

## 3. Experimental

### 3.1. General Methods

All the enzymes were purchased from the Sigma-Aldrich Chemical Company and stored at 4 °C. All solvents used were HPLC or ACS grade. THF and Et_2_O were distilled from Na/benzophenone. CH_2_Cl_2_ was distilled from CaH_2_. Nuclear magnetic resonance (NMR) spectra were recorded on a Varian Gemini 200 or Bruker DPX 300 spectrometer at operating frequencies of 200 and 300 MHz (^1^H-NMR) or 50 and 75 MHz (^13^C-NMR). The ^1^H-NMR chemical shifts are reported in ppm relative to TMS peak. Data are reported as follows: chemical shift (δ), multiplicity (s = singlet, d = doublet, t = triplet, q = quartet, m = multiplet), coupling constant (*J*) in Hertz and integrated intensity. The ^13^C-NMR chemical shifts are reported in ppm relative to CDCl_3_ signal. Infrared spectra were recorded from KBr discs on a Bomem Michelson model 101 FTIR spectrometer. Absorption maxima (ν_max_) are reported in wavenumbers (cm^−1^). Low-resolution mass spectra were obtained on a GC/MS Shimadzu spectrometer, operating at 70 eV. High-resolution mass spectra (HRMS) were acquired using a Bruker Daltonics MicroTOF instrument, operating electrospray ionization (ESI) mode. Gas chromatography (GC) analysis for measurement of enantiomeric excess was obtained using a Shimadzu 17-A Gas Chromatograph, Flame Ionization Detector (FID), and a chiral column Varian CP-Chirasil-DEX CB β-ciclodextrin (25 m × 0.32 mm × 0.25 μm). Temperature of the detector and injector was 250 °C; oven temperature: from 100 °C to 150 °C, 1 °C/min; flow = 80 kPa, H_2_ as gas carrier.

### 3.2. Synthetic Procedures

#### 3.2.1. General Procedure for the Preparation of 1-(Bromophenyl)ethanols **1a–c** [35,47]

Ketone (10 mmol, 1.99 g) was added to a 250 mL round-bottomed flask containing ethanol (20 mL) cooled at 0 °C, followed by the addition of NaBH_4_ (11 mmol, 0.42 mg) in small portions. The mixture was stirred for 30 min at 0 °C and for 2.5 h at room temperature. The ethanol was evaporated under reduced pressure and H_2_O (20 mL) was added to the organic residue. The mixture was treated with aqueous acid solution (1 M HCl) until pH 6, and extracted with CH_2_Cl_2_ (3 × 20 mL). Organic phase was dried over anhydrous MgSO_4_, and the solvent was evaporated under reduced pressure. The crude product was purified by column chromatography using *n*-hexane/ethyl acetate (8:2) as eluent. All spectral data of compounds **1a–c** were in agreement with those described in the literature [48].

#### 3.2.2. General Procedure for the Preparation of 1-(Bromophenyl)propanols **1d–f** [49]

To a 100 mL, two-necked, round-bottom flask fitted with a reflux condenser and containing a suspension of magnesium (15 mmol, 0.36 g) in Et_2_O (4 mL), a solution of bromoethane (15 mmol, 1.63 g, 1.12 mL) in Et_2_O (12 mL) was added dropwise under N_2_ atmosphere. The mixture was refluxed for 30 min. Then the solution of ethylmagnesium bromide was cooled to −5 °C and a solution of the appropriate brom-benzaldehyde (10 mmol, 1.85 g) in Et_2_O (8 mL) was added dropwise. The mixture was stirred for 1 h at r.t., poured on ice, and acidified with 6 M HCl. The organic layer was separated, and the aqueous layer was extracted with Et_2_O (3 × 5 mL). The organic solutions were combined, washed with 10% NaHCO_3_ solution (2 × 5 mL), H_2_O (2 × 5 mL), brine (2 × 5 mL), and dried over anhydrous MgSO_4_. The crude product was purified by column chromatography using *n*-hexane/ethyl acetate (9:1) as eluent. All spectral data of the compounds **1d–f** were in agreement with those described in the literature [42].

#### 3.2.3. General Procedure for the Preparation of Aryltrimethylsilyl Chiral Alcohols **2a–f** [11,50]

A 50 mL, two-necked, round-bottom flask under N_2_ atmosphere was charged with a solution of the appropriate alcohol **1a–f** (5 mmol) in THF (15 mL). The mixture was cooled to −70 °C and, a solution of *n*-butyllithium (12.5 mmol, 1.6 M in hexanes, 7.80 mL) was added dropwise. The mixture was stirred at −70 °C for 2 h. Trimethylsilyl chloride (14 mmol, 1.52 g, 1.78 mL) was added, and after 30 min at −70 °C, the solution was allowed to warm to room temperature and stirred overnight. The reaction was quenched by the addition of aqueous acid solution (1 M HCl, 15 mL) and stirred for 1 hour. The organic layer was separated and the aqueous layer extracted with ethyl acetate (3 × 10 mL). The combined organic layers were washed with saturated solution of NaHCO_3_ (2 × 25 mL) and brine (2 × 25 mL), dried over anhydrous MgSO_4_ and concentrated *in vacuo*. The residue was purified by column chromatography using *n*-hexane/ethyl acetate (9:1) as eluent.

*1-(2-(Trimethylsilyl)phenyl)ethanol *(**2a**). Yield: 606 mg (62%). White solid. ^1^H-NMR (200 MHz, CDCl_3_) δ = 0.35 (s, 9H), 1.49 (d, *J *= 6.0 Hz, 3H), 5.11 (q, *J *= 6.0 Hz, 1H), 7.23–7.64 (m, 4H). ^13^C-NMR (50 MHz, CDCl_3_): δ = 0.85, 25.38, 69.84, 125.32, 127.21, 130.07, 134.42, 137.06, 151.65. FT-IR (KBr) νmax = 3284, 3058, 2956, 1930, 1630, 1432, 1247, 1099, 1072, 1000, 848, 731 cm^−1^. LRMS (EI) *m/z *(%) = 179(15), 161(100), 145(62), 133(21), 119(8), 104(12), 91(10), 75(33), 59(9), 43(49). HRMS (ESI): Calculated for C_11_H_18_OSiNa [M + Na]^+^ = 217.1025; found: 217.1018.

*1-(3-(Trimethylsilyl)phenyl)ethanol *(**2b**). Yield: 702 mg (72%). Colourless oil. ^1^H-NMR (200 MHz, CDCl_3_) δ = 0.27 (s, 9H), 1.50 (d, *J *= 6.0 Hz, 3H), 4.86 (q, *J *= 6.0 Hz, 1H), 7.30–7.52 (m, 4H). ^13^C-NMR (50 MHz, CDCl_3_): δ = −0.93, 25.32, 70.84, 126.08, 128.09, 130.46, 132.75, 141.02, 145.05. FT-IR (KBr) νmax = 3343, 3048, 2956, 1594, 1450, 1248, 1098, 858, 837, 753 cm^−1^. LRMS (EI) *m/z *(%) = 194(7) [M]^+^, 179(100), 161(33), 145(8), 135(15), 119(10), 104(11), 91(8), 75(54), 59(9), 43(60). HRMS (ESI): Calculated for C_11_H_18_OSiNa [M + Na]^+^ = 217.1025; found: 217.1015.

*1-(4-(Trimethylsilyl)phenyl)ethanol *(**2c**). Yield: 743 mg (76%). White waxy solid. ^1^H-NMR (200 MHz, CDCl_3_) δ = 0.30 (s, 9H), 1.51 (d, *J *= 6.0 Hz, 3H), 4.86 (q, *J *= 6.0 Hz, 1H), 7.37 (d, *J *= 8.0 Hz, 2H), 7.53 (d, *J *= 8.0 Hz, 2H). ^13^C-NMR (50 MHz, CDCl_3_): δ = −0.94, 25.20, 70.57, 124.96, 133.75, 139.83, 146.53. FT-IR (KBr) νmax = 3323, 3068, 2955, 1918, 1601, 1410, 1247, 1082, 1071, 1003, 857, 822 cm^−1^. LRMS (EI) *m/z *(%) = 194(8) [M]^+^, 179(100), 163(6), 151(4), 135(8), 119(7), 105(12), 91(7), 73(23), 59(3), 43(38). HRMS (ESI): Calculated for C_11_H_18_OSiNa [M + Na]^+^ = 217.1025; found: 217.1019.

*1-(2-(Trimethylsilyl)phenyl)propanol *(**2d**). Yield: 673 mg (65%). Colourless oil. ^1^H-NMR (300 MHz, CDCl3) δ = 0.34 (s, 9H), 1.01 (t, *J* = 6.0 Hz, 3H), 1.69–1.88 (m, 2H), 4.83 (m, 1H), 7.23–7.55 (m, 4H). ^13^C-NMR (75 MHz, CDCl_3_): δ = 0.96, 11.01, 32.11, 75.22, 125.57, 127.17, 129.94, 134.53, 137.63, 150.67. FT-IR (KBr) νmax = 3387, 3056, 2959, 1590, 1459, 1250, 1105, 968, 838, 730 cm^−1^. LRMS (EI) *m/z *(%) = 193(16), 175(87), 163(100), 145(18), 135(12), 117(15), 105(14), 91(12), 75(53), 59(94), 45(39). HRMS (ESI): Calculated for C_12_H_20_OSiNa [M + Na]^+^ = 231.1181; found: 231.1175.

*1-(3-(Trimethylsilyl)phenyl)propanol *(**2e**). Yield: 725 mg (70%). Colourless oil. ^1^H-NMR (300 MHz, CDCl3) δ = 0.27 (s, 9H), 0.92 (t, *J* = 6.0 Hz, 3H), 1.68–1.86 (m, 2H), 4.55 (t, *J* = 6.0 Hz, 1H), 7.30–7.47 (m, 4H). ^13^C-NMR (75 MHz, CDCl_3_): δ = −0.94, 10.40, 32.06, 76.38, 126.60, 127.94, 131.03, 132.67, 140.75, 143.92. FT-IR (KBr) νmax = 3351, 3052, 2958, 1586, 1441, 1248, 1121, 860, 837, 753 cm^−1^. LRMS (EI) *m/z *(%) = 208(4) [M]^+^, 193(32), 179(100), 163(10), 145(4), 135(10), 119(7), 105(10), 91(9), 73(94), 59(26), 43(31). HRMS (ESI): Calculated for C_12_H_20_OSiNa [M + Na]^+^ = 231.1181; found: 231.1171.

*1-(4-(Trimethylsilyl)phenyl)propanol *(**2f**). Yield: 706 mg (68%). White solid. ^1^H-NMR (300 MHz, CDCl_3_) δ = 0.26 (s, 9H), 0.30 (t, *J* = 6.0 Hz, 3H), 1.70–1.85 (m, 2H), 4.57 (t, *J* = 6.0 Hz, 1H), 7.31 (d, *J *= 9.0 Hz, 2H), 7.49 (d, *J *= 9.0 Hz, 2H). ^13^C-NMR (75 MHz, CDCl_3_): δ = −0.92, 10.38, 31.97, 76.19, 125.53, 133.64, 139.81, 145.36. FT-IR (KBr) νmax = 3323, 3068, 2957, 1598, 1453, 1401, 1248, 1111, 1094, 853, 837 cm^−1^. LRMS (EI) *m/z *(%) = 208(3) [M]^+^, 193(51), 179(100), 163(14), 151(7), 135(14), 119(8), 105(11), 91(13), 73(76), 59(8), 43(28). HRMS (ESI): Calculated for C_12_H_20_OSiNa [M + Na]^+^ = 231.1181; found: 231.1176.

#### 3.2.4. General Procedure for the Preparation of Aryltrimethylsilyl Chiral Acetates **3a–f** [51]

To a 25 mL round-bottom flask containing a solution of the appropriate aryltrimethylsilyl chiral alcohol (0.5 mmol) in CH_2_Cl_2_ (5 mL), was added acetic anhydride (0.6 mmol, 0.06 g, 56.3 µL), pyridine (2 mmol, 0.16 g, 161.5 µL) and DMAP (0.1 mmol, 12.2 mg), sequentially. The mixture was stirred overnight at r.t. Then the solvent was evaporated under reduced pressure and ethyl acetate (15 mL) was added to the organic residue. The organic layer was washed with aq. 1 M CuSO_4_ (2 × 10 mL), brine (2 × 10 mL), dried over anhydrous MgSO_4_ and concentrated *in vacuo*. The residue was purified by column chromatography using *n*-hexane/ethyl acetate (9:1) as eluent.

*1-(2-(Trimethylsilyl)phenyl)ethyl acetate *(**3a**). Yield: 112 mg (95%). Colourless oil. ^1^H-NMR (200 MHz, CDCl_3_) δ = 0.36 (s, 9H), 1.50 (d, *J *= 6.0 Hz, 3H), 2.06 (s, 3H), 6.06 (q, *J *= 6.0 Hz, 1H), 7.24–7.55 (m, 4H). ^13^C-NMR (50 MHz, CDCl_3_): δ = 0.63, 21.61, 23.62, 72.43, 125.97, 127.38, 129.77, 134.48, 137.29, 147.73, 170.37. FT-IR (KBr) νmax = 3024, 2960, 1740, 1599, 1436, 1237, 1019, 1037, 838 cm^−1^. LRMS (EI) *m/z *(%) = 221(5), 193(3), 177(3), 161(30), 145(25), 133(8), 117(67), 103(6), 91(3), 75(19), 59(7), 43(100). HRMS (ESI): Calculated for C_13_H_20_O_2_SiNa [M + Na]^+^ = 259.1130; found: 259.1128.

*1-(3-(Trimethylsilyl)phenyl)ethyl acetate *(**3b**). Yield: 115 mg (98%). Colourless oil. ^1^H-NMR (200 MHz, CDCl_3_) δ = 0.27 (s, 9H), 1.53 (d, *J *= 6.0 Hz, 3H), 2.07 (s, 3H), 5.85 (q, *J *= 6.0 Hz, 1H), 7.33–7.48 (m, 4H). ^13^C-NMR (50 MHz, CDCl_3_): δ = −0.95, 21.56, 22.44, 72.70, 126.72, 128.04, 131.15, 133.11, 140.90, 140.99, 170.50. FT-IR (KBr) νmax = 3050, 2956, 1739, 1587, 1448, 1247, 1065, 1024, 839 cm^−1^. LRMS (EI) *m/z *(%) = 236(4) [M]^+^, 221(1), 193(11), 177(4), 161(28), 135(4), 117(30), 103(5), 91(2), 75(33), 59(4), 43(100). HRMS (ESI): Calculated for C_13_H_20_O_2_SiNa [M + Na]^+^ = 259.1130; found: 259.1125.

*1-(4-(Trimethylsilyl)phenyl)ethyl acetate *(**3c**). Yield: 114 mg (97%). Colourless oil. ^1^H-NMR (200 MHz, CDCl_3_) δ = 0.30 (s, 9H), 1.52 (d, *J *= 6.0 Hz, 3H), 2.07 (s, 3H), 5.86 (q, *J *= 6.0 Hz, 1H), 7.32 (d, *J *= 8.0 Hz, 2H), 7.49 (d, *J *= 8.0 Hz, 2H). ^13^C-NMR (50 MHz, CDCl_3_): δ = −0.98, 21.51, 22.29, 72.48, 125.60, 133.71, 140.31, 142.30, 170.50. FT-IR (KBr) νmax = 3020, 2955, 1738, 1602, 1447, 1241, 1063, 1022, 850 cm^−1^. LRMS (EI) *m/z *(%) = 236(2) [M]^+^, 221(7), 194(8), 177(4), 161(14), 151(14), 133(4), 119(11), 103(7), 91(4), 75(19), 59(5), 43(100). HRMS (ESI): Calculated for C_13_H_20_O_2_SiNa [M + Na]^+^ = 259.1130; found: 259.1131.

*1-(2-(Trimethylsilyl)phenyl)propyl acetate *(**3d**). Yield: 121 mg (97%). Colourless oil. ^1^H-NMR (300 MHz, CDCl_3_) δ = 0.38 (s, 9H), 0.96 (t, *J* = 6.0 Hz, 3H), 1.71–1.91 (m, 2H), 2.07 (s, 3H), 5.93 (t, *J* = 6 Hz, 1H), 7.23–7.51 (m, 4H). ^13^C-NMR (75 MHz, CDCl_3_): δ = 0.78, 10.59, 21.44, 31.16, 77.09, 125.88, 127.23, 129.53, 134.63, 137.61, 147.11, 170.54. FT-IR (KBr) νmax = 3058, 2967, 1738, 1591, 1434, 1372, 1235, 1085, 1046, 840 cm^−1^. LRMS (EI) *m/z *(%) = 235(1), 207(1), 175(4), 163(11), 145(6), 135(4), 117(55), 105(4), 91(3), 75(15), 59(25), 43(100). HRMS (ESI): Calculated for C_14_H_22_O_2_SiNa [M + Na]^+^ = 273.1287; found: 273.1283.

*1-(3-(Trimethylsilyl)phenyl)propyl acetate* (**3e**). Yield: 118 mg (95%). Colourless oil. ^1^H-NMR (300 MHz, CDCl_3_) δ = 0.27 (s, 9H), 0.89 (t, *J* = 6.0 Hz, 3H), 1.76–1.97 (m, 2H), 2.08 (s, 3H), 5.68 (t, *J* = 6 Hz, 1H), 7.31–7.46 (m, 4H). ^13^C-NMR (75 MHz, CDCl_3_): δ = −0.94, 10.19, 21.46, 29.62, 77.72, 127.13, 127.93, 131.64, 133.04, 139.88, 140.83, 170.61. FT-IR (KBr) νmax = 3050, 2957, 1740, 1459, 1372, 1237, 1085, 1020, 839 cm^−1^. LRMS (EI) *m/z *(%) = 250(4) [M]^+^, 235(1), 221(3), 207(9), 175(20), 163(8), 135(4), 117(34), 105(4), 91(4), 75(32), 59(8), 43(100). HRMS (ESI): Calculated for C_14_H_22_O_2_SiNa [M + Na]^+^ = 273.1287; found: 273.1288.

*1-(4-(Trimethylsilyl)phenyl)propyl acetate *(3**f**). Yield: 119 mg (96%). Colourless oil. ^1^H-NMR (200 MHz, CDCl_3_) δ = 0.25 (s, 9H), 0.89 (t, *J* = 6.0 Hz, 3H), 1.73–2.00 (m, 2H), 2.07 (s, 3H), 5.65 (t, *J *= 6 Hz, 1H), 7.28 (d, *J *= 8.0 Hz, 2H), 7.47 (d, *J *= 8.0 Hz, 2H). ^13^C-NMR (50 MHz, CDCl_3_): δ = −0.97, 10.14, 21.42, 29.43, 77.54, 126.05, 133.58, 140.17, 141.20, 170.61. FT-IR (KBr) νmax = 3024, 2960, 1740, 1599, 1436, 1237, 1085, 1019, 838 cm^−1^. LRMS (EI) *m/z *(%) = 250(5) [M]^+^, 235(4), 221(8), 208(15), 179(23), 165(9), 135(4), 117(33), 105(6), 91(4), 75(27), 59(7), 43(100). HRMS (ESI): Calculated for C_14_H_22_O_2_SiNa [M + Na]^+^ = 273.1287; found: 273.1282. 

### 3.3. Determination of the Enantiomeric Excess (ee)

The enantiomeric excesses of the aryltrimethylsilyl chiral alcohols **2a–f** and esters **3a–f** were measured by chiral GC analysis. The analysis was carried out on Varian CP-Chirasil-DEX CB β-ciclodextrin (25 m × 0.32 mm × 0.25μm). Temperature of the detector and injector was 250 °C; oven temperature: from 100 °C to 150 °C, 1 °C/min; flow = 80 kPa, H_2_ as gas carrier.

Retention times (*t*_R_):

(**2a**): *t*_R_ = 19.8 min for (*S*) and *t*_R_ = 20.9 min for (*R*);
(**2b**): *t*_R_ = 19.6 min for (*S*) and *t*_R_ = 20.1 min for (*R*);
(**2c**): *t*_R_ = 26.5 min for (*S*) and *t*_R_ = 25.6 min for (*R*);
(**2d**): *t*_R_ = 21.6 min for (*S*) and *t*_R_ = 22.6 min for (*R*);
(**2e**): *t*_R_ = 25.0 min for (*S*) and *t*_R_ = 24.4 min for (*R*);
(**2f**): *t*_R_ = 31.2 min for (*S*) and *t*_R_ = 30.3 min for (*R*);
(**3a**): *t*_R_ = 16.0 min for (*S*) and *t*_R_ = 16.2 min for (*R*);
(**3b**): *t*_R_ = 16.2 min for (*S*) and *t*_R_ = 16.8 min for (*R*);
(**3c**): *t*_R_ = 23.7 min for (*S*) and *t*_R_ = 25.9 min for (*R*);
(**3d**): *t*_R_ = 63.4 min for (*S*) and *t*_R_ = 63.6 min for (*R*) (oven: 50 °C to 150 °C, 1 °C/min);
(**3e**): *t*_R_ = 22.0 min for (*S*) and *t*_R_ = 20.4 min for (*R*);
(**3f**): *t*_R_ = 26.3 min for (*S*) and *t*_R_ = 27.1 min for (*R*);

### 3.4. Enzymatic Procedures

#### 3.4.1. General Procedure for Small Scale Enzymatic Reactions

A 1.5 mL Eppendorf tube (2 mL) was charged with the appropriate aryltrimethylsilyl chiral alcohol **2a–f** (0.05 mmol), hexanes (HPLC grade, 200 µL), CAL-B (enzyme amount as indicated in Table 1, Table 2 and Table 3) and vinyl acetate (2.2 equiv., 0.11 mmol, 10 μL). The reaction mixture was sealed and stirred on a thermomixer (30 °C, 700 rpm) until the appropriate time. After that, the mixture was filtered and the solvent evaporated under reduced pressure.

#### 3.4.2. General Procedure for Preparative-Scale Enzymatic Reactions

A 10 mL glass tube was charged with the appropriate aryltrimethylsilyl chiral alcohol **2a–f** (1 mmol), hexanes (HPLC grade, 4 mL), CAL-B (enzyme amount as indicated on Table 4) and vinyl acetate (2.2 mmol, 203 µL). The reaction mixture was sealed and stirred on a thermomixer (30 °C, 700 rpm) until the appropriate time. After that, the mixture was filtered and the solvent evaporated under reduced pressure. The purification of alcohol **2** and acetate **3** was performed by column chromatography using *n*-hexane/ethyl acetate (9:1) as eluent.

(*S*)-**2b**: isolated yield = 44%; enantiomeric excess > 99%; [α]_D_^20^ = -37.0 (*c *= 1.0; CHCl_3_).
(*R*)-**3b**: isolated yield = 49%; enantiomeric excess > 99%; [α]_D_^20^ = +70.8 (*c *= 1.0; CHCl_3_).
(*S*)-**2c**: isolated yield = 31%; enantiomeric excess > 99%; [α]_D_^20^ = -31.2 (*c *= 1.0; CHCl_3_).
(*R*)-**3c**: isolated yield = 46%; enantiomeric excess > 99%; [α]_D_^20^ = +93.9 (*c *= 1.0; CHCl_3_).
(*S*)-**2e**: isolated yield = 38%; enantiomeric excess > 99%; [α]_D_^20^ = -32.9 (*c *= 1.0; CHCl_3_).
(*R*)-**3e**: isolated yield = 49%; enantiomeric excess > 99%; [α]_D_^20^ = +76.8 (*c *= 1.0; CHCl_3_).
(*S*)-**2f**: isolated yield = 39%; enantiomeric excess > 99%; [α]_D_^20^ = -34.1 (*c *= 1.0; CHCl_3_).
(*R*)-**3f**: isolated yield = 34%; enantiomeric excess > 99%; [α]_D_^20^ = +91.3 (*c *= 1.0; CHCl_3_).

### 3.5. Determination of the Absolute Configuration

#### 3.5.1. General Procedure for Transformation of Enantioenriched Aryltrimethylsilyl Chiral Alcohols **2b**, **2c**, **2e** and **2f** to Their Bromide Derivatives [52]

To a solution of the appropriate enantioenriched aryltrimethylsilyl chiral alcohol (0.05 mmol) dissolved in MeOH (1.0 mL) was added KBr (0.05 mmol, 6 mg) and NCS (0.05 mmol, 7 mg). The mixture was stirred for 20 min at 60 °C. The reaction was quenched with H_2_O (2 mL) and extracted with CH_2_Cl_2_ (2 × 5 mL). The organic layer was washed with brine (2 × 2 mL), dried over anhydrous MgSO_4_ and concentrated *in vacuo*. The residue was purified by column chromatography using *n*-hexane/ethyl acetate (9:1) as eluent.

#### 3.5.2. Determination of Absolute Configuration of Enantioenriched Aryltrimethylsilyl Chiral Alcohols **2b**, **2c**, **2e** and **2f**

The enantioenriched alcohols **1b**, **1c**, **1e** and **1f** obtained from the bromodesilylation reactions (section 3.4.1) were submitted to chiral GC and HPLC analysis, and the values found for retention times were in agreement with enantiopure samples and those described in the literature for enantioenriched compounds [42,43,44,45]. The absolute configuration of compounds **2b**, **2c**, **2e** and **2f** were attributed by analogy of those found to its bromide derivatives.

## 4. Conclusions

An extensive study of lipase-catalyzed kinetic resolution of aryltrimethylsilyl chiral alcohols through an enantioselective transesterification was described. The best lipase source found for kinetic resolution of these silicon-containing compounds was *Pseudomonas cepacia* (Amano PS-C II). Different enzyme/substrate ratios were evaluated and a very small amount of lipase proved to be satisfactory for the kinetic resolution in most cases. The kinetic resolution of *o*-substituted phenyl chiral alcohols did not occur under the conditions evaluated. The reason for this surprisingly results is still opened for further studies, but a possible explanation is that a pseudo-pentacoordinated species generated by a non-bonded Si**^…^**O attraction, should create a steric hindrance that made the kinetic resolution more difficult to occur. On the other hand, perfect kinetic resolutions were achieved for the *m*- and *p*-substituted phenyl chiral alcohols, in which (*S*)-alcohols and (*R*)-acetates were obtained in high yields (up to 49%) and excellent enantiomeric excesses (>99%).
